# *catena*-Poly[[[diaqua­(nitrato-κ^2^ *O*,*O*′)cerium(III)]-bis­[μ-2-(4-hy­droxy­phen­yl)acetato]-κ^3^ *O*,*O*′:*O*;κ^3^ *O*:*O*,*O*′] mono­hydrate]

**DOI:** 10.1107/S1600536810047239

**Published:** 2010-11-20

**Authors:** Hang-Ming Guo

**Affiliations:** a Jinhua College of Vocation and Technology, Jinhua, Zhejiang 321017, People’s Republic of China

## Abstract

In the title compound, {[Ce(C_8_H_7_O_3_)_2_(NO_3_)(H_2_O)_2_]·H_2_O}_*n*_, the Ce^III^ ion is coordinated by eight O atoms from four 2-(4-hy­droxy­phen­yl)acetate (HPAA) ligands, two O atoms from the chelating nitrate anion and two water mol­ecules in a distorted bis-capped quadrangular prismatic geometry. The HPAA ligands coordinate in a bridging tridentate mode. In the crystal, inter­molecular O—H⋯O hydrogen bonds form a three-dimensional network which consolidates the packing.

## Related literature

For the crystal structures of related carb­oxy­lic metal-organic complexes, see: Liu *et al.* (2010 ▶); Fang & Zhang (2006 ▶); Wang *et al.* (2008 ▶, 2010 ▶). 
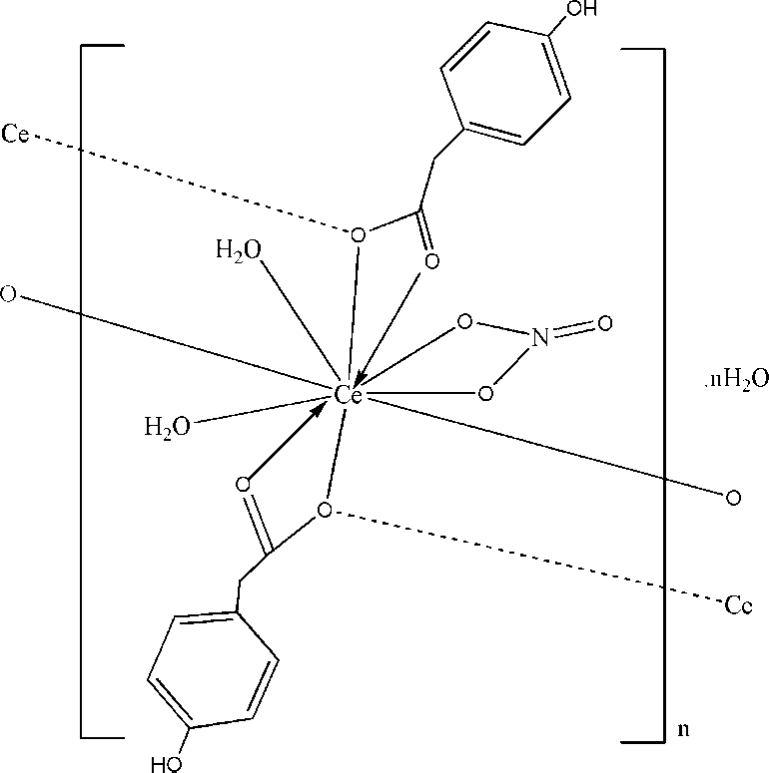


## Experimental

### 

#### Crystal data

[Ce(C_8_H_7_O_3_)_2_(NO_3_)(H_2_O)_2_]·H_2_O
*M* *_r_* = 558.45
Triclinic, 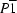

*a* = 8.1151 (3) Å
*b* = 9.8048 (4) Å
*c* = 13.2396 (5) Å
α = 92.120 (2)°
β = 90.829 (2)°
γ = 112.550 (2)°
*V* = 971.76 (6) Å^3^
*Z* = 2
Mo *K*α radiation
μ = 2.41 mm^−1^
*T* = 296 K
0.14 × 0.14 × 0.03 mm

#### Data collection

Bruker APEXII diffractometer
Absorption correction: multi-scan (*SADABS*; Sheldrick, 1996 ▶) *T*_min_ = 0.716, *T*_max_ = 0.935
15535 measured reflections
4492 independent reflections
4133 reflections with *I* > 2σ(*I*)
*R*_int_ = 0.026

#### Refinement

*R*[*F* ^2^ > 2σ(*F* ^2^)] = 0.020
*wR*(*F* ^2^) = 0.050
*S* = 0.98
4492 reflections
289 parameters
9 restraints
H atoms treated by a mixture of independent and constrained refinement
Δρ_max_ = 0.72 e Å^−3^
Δρ_min_ = −0.36 e Å^−3^

### 

Data collection: *APEX2* (Bruker, 2006 ▶); cell refinement: *SAINT* (Bruker, 2006 ▶); data reduction: *SAINT*; program(s) used to solve structure: *SHELXS97* (Sheldrick, 2008 ▶); program(s) used to refine structure: *SHELXL97* (Sheldrick, 2008 ▶); molecular graphics: *SHELXTL* (Sheldrick, 2008 ▶); software used to prepare material for publication: *SHELXTL*.

## Supplementary Material

Crystal structure: contains datablocks I, global. DOI: 10.1107/S1600536810047239/cv2794sup1.cif

Structure factors: contains datablocks I. DOI: 10.1107/S1600536810047239/cv2794Isup2.hkl

Additional supplementary materials:  crystallographic information; 3D view; checkCIF report

## Figures and Tables

**Table 1:** Hydrogen-bond geometry (Å, °)

*D*—H⋯*A*	*D*—H	H⋯*A*	*D*⋯*A*	*D*—H⋯*A*
O1*W*—H1*WA*⋯O2^i^	0.85 (2)	1.90 (2)	2.693 (2)	156 (3)
O1*W*—H1*WB*⋯O7^ii^	0.82 (2)	2.46 (2)	3.197 (3)	150 (3)
O1*W*—H1*WB*⋯O8^ii^	0.82 (2)	2.51 (2)	3.272 (3)	156 (3)
O2*W*—H2*WA*⋯O6^iii^	0.83 (2)	1.91 (2)	2.721 (2)	165 (3)
O2*W*—H2*WB*⋯O4^iv^	0.84 (2)	1.95 (2)	2.783 (3)	175 (3)
O3*W*—H3*WA*⋯O4^i^	0.85 (2)	2.58 (2)	3.352 (4)	152 (3)
O4—H4*A*⋯O1^v^	0.82	1.83	2.649 (3)	173

Symmetry codes: (i) 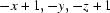; (ii) 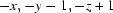; (iii) 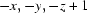; (iv) 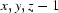; (v) 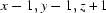.

**Fig. 1.**
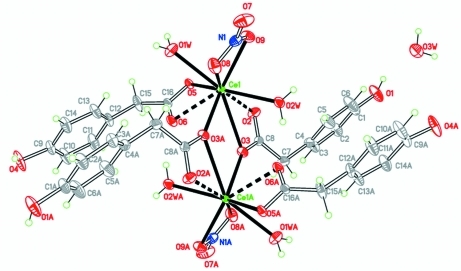
A portion of the polymeric chain of (I), showing the atom-labeling scheme [symmetry code: (A) -x, -y, 1-z]. Displacement ellipsoids are drawn at the 30% probability level.

## supplementary crystallographic information

### Comment

The design and synthesis of carboxylic mental-organic complexes have attracted an interest owing to their potential practical applications exhibiting fluorescence and magnetism (Wang *et al.*, 2008, 2010; Fang *et al.*, 2006). In a continuation of our structural studies of such compounds  (Liu *et al.*, 2010), we report here the crystal structure of the title compound (I) - a new cerium^III^ complex with the *p*-hydroxyphenylacetato ligands.

In (I), each Ce^III^ ion is coordinated by eight O atoms from four 4–hydroxyphenylacetato (HPAA) ligands, two O atoms from nitrate anion and two water molecules in a distorted bis-capped quadrangular prism geometry. The HPAA ligands coordinate in bridging tridentate mode (Fig.1). The Ce—O(HPAA) bond lengths range from 2.4914 (14) to 2.7245 (15) Å. The Ce—O(water) bond lengths range from 2.5444 (16) to Å-2.5463 (16) Å.

In the crystal structure, intermolecular O—H···O hydrogen bonds (Table 1) form three-dimensional network which concolidate the packing.

### Experimental

All reagents and solvents used were of commercially available quality and without purification. *p*-Hydroxyphenylacetic acid (0.456 g, 3 mmol) and sodium hydroxide (0.12 g, 3 mmol) were mixed together in water(10 ml), then Ce(NO_3_)_3_.6H_2_O(0.434 g, 1 mmol) dissolved in water (10 ml) was added into the above solution, after stirred for an hour. After filtration, the filtrate was allowed to stand at room temperature, and single crystals suitable for *X*–ray work were obtained after a week.

### Refinement

All H atoms attached to C atoms and *O*(hydroxyl) atom were fixed geometrically and treated as riding with C—H = 0.97 Å (methylene) or 0.93 Å (aromatic) and O—H = 0.82 Å with *U*_iso_(H) = 1.2*U*_eq_(C) or *U*_iso_(H) = 1.5*U*_eq_(O). H atoms of water molecule were located in a difference Fourier map and included in the subsequent refinement using restraints (O—H= 0.82 (1)Å and H···H= 1.39 (2) Å) with *U*_iso_(H) = 1.5*U*_eq_(O). In the last cycles of refinement they were treated as riding on their parent O atom.

### Crystal data

**Table:**

[Ce(C_8_H_7_O_3_)_2_(NO_3_)(H_2_O)_2_]·H_2_O	*Z* = 2
*M**_r_* = 558.45	*F*(000) = 554
Triclinic, *P*1	*D*_x_ = 1.909 Mg m^−^^3^
Hall symbol: -P 1	Mo *K*α radiation, λ = 0.71073 Å
*a* = 8.1151 (3) Å	Cell parameters from 8276 reflections
*b* = 9.8048 (4) Å	θ = 2.3–27.7°
*c* = 13.2396 (5) Å	µ = 2.41 mm^−^^1^
α = 92.120 (2)°	*T* = 296 K
β = 90.829 (2)°	Block, colourless
γ = 112.550 (2)°	0.14 × 0.14 × 0.03 mm
*V* = 971.76 (6) Å^3^	

### Data collection

**Table:**

Bruker APEXII diffractometer	4492 independent reflections
Radiation source: fine-focus sealed tube	4133 reflections with *I* > 2σ(*I*)
graphite	*R*_int_ = 0.026
ω scans	θ_max_ = 27.7°, θ_min_ = 2.3°
Absorption correction: multi-scan (*SADABS*; Sheldrick, 1996)	*h* = −10→10
*T*_min_ = 0.716, *T*_max_ = 0.935	*k* = −12→12
15535 measured reflections	*l* = −16→17

### Refinement

**Table:**

Refinement on *F*^2^	Primary atom site location: structure-invariant direct methods
Least-squares matrix: full	Secondary atom site location: difference Fourier map
*R*[*F*^2^ > 2σ(*F*^2^)] = 0.020	Hydrogen site location: inferred from neighbouring sites
*wR*(*F*^2^) = 0.050	H atoms treated by a mixture of independent and constrained refinement
*S* = 0.98	*w* = 1/[σ^2^(*F*_o_^2^) + (0.0337*P*)^2^ + 0.0249*P*] where *P* = (*F*_o_^2^ + 2*F*_c_^2^)/3
4492 reflections	(Δ/σ)_max_ = 0.001
289 parameters	Δρ_max_ = 0.72 e Å^−^^3^
9 restraints	Δρ_min_ = −0.35 e Å^−^^3^

### Special details

**Table:**

Geometry. All e.s.d.'s (except the e.s.d. in the dihedral angle between two l.s. planes) are estimated using the full covariance matrix. The cell e.s.d.'s are taken into account individually in the estimation of e.s.d.'s in distances, angles and torsion angles; correlations between e.s.d.'s in cell parameters are only used when they are defined by crystal symmetry. An approximate (isotropic) treatment of cell e.s.d.'s is used for estimating e.s.d.'s involving l.s. planes.
Refinement. Refinement of *F*^2^ against ALL reflections. The weighted *R*-factor *wR* and goodness of fit *S* are based on *F*^2^, conventional *R*-factors *R* are based on *F*, with *F* set to zero for negative *F*^2^. The threshold expression of *F*^2^ > σ(*F*^2^) is used only for calculating *R*-factors(gt) *etc*. and is not relevant to the choice of reflections for refinement. *R*-factors based on *F*^2^ are statistically about twice as large as those based on *F*, and *R*- factors based on ALL data will be even larger.

### Fractional atomic coordinates and isotropic or equivalent isotropic displacement parameters (Å^2^)

**Table:**

	*x*	*y*	*z*	*U*_iso_*/*U*_eq_	
Ce1	0.208699 (13)	−0.089222 (12)	0.466072 (8)	0.02011 (5)	
N1	0.0546 (3)	−0.3859 (2)	0.33905 (16)	0.0335 (4)	
O1W	0.2726 (2)	−0.28503 (19)	0.56174 (16)	0.0402 (4)	
H1WA	0.379 (2)	−0.277 (3)	0.573 (2)	0.060*	
H1WB	0.214 (3)	−0.364 (2)	0.587 (2)	0.060*	
O1	0.7474 (3)	0.5608 (2)	0.07849 (16)	0.0632 (6)	
H1A	0.7371	0.4920	0.0384	0.095*	
O2	0.3683 (2)	0.18203 (18)	0.42823 (15)	0.0372 (4)	
O2W	0.0678 (2)	−0.0555 (2)	0.30083 (12)	0.0346 (4)	
H2WA	−0.019 (3)	−0.031 (3)	0.304 (2)	0.052*	
H2WB	0.055 (4)	−0.101 (3)	0.2447 (16)	0.052*	
O3	0.09817 (19)	0.14048 (17)	0.47414 (12)	0.0290 (3)	
O3W	0.7347 (4)	0.4032 (3)	−0.09596 (17)	0.0650 (6)	
H3WA	0.801 (5)	0.363 (4)	−0.120 (3)	0.098*	
H3WB	0.721 (6)	0.448 (4)	−0.144 (2)	0.098*	
O4	0.0288 (3)	−0.1882 (3)	1.10768 (14)	0.0645 (7)	
H4A	−0.0557	−0.2660	1.0935	0.097*	
O5	0.4853 (2)	0.03842 (17)	0.59596 (11)	0.0272 (3)	
O6	0.2196 (2)	−0.0107 (2)	0.65299 (12)	0.0358 (4)	
O7	−0.0071 (3)	−0.4974 (2)	0.2852 (2)	0.0635 (7)	
O8	−0.0245 (2)	−0.3651 (2)	0.41577 (15)	0.0475 (5)	
O9	0.2019 (2)	−0.28460 (19)	0.32163 (14)	0.0404 (4)	
C1	0.6258 (3)	0.5107 (3)	0.1536 (2)	0.0408 (6)	
C2	0.6439 (4)	0.6015 (3)	0.2378 (2)	0.0428 (6)	
H2A	0.7371	0.6938	0.2434	0.051*	
C3	0.5227 (3)	0.5555 (3)	0.3150 (2)	0.0390 (6)	
H3A	0.5352	0.6178	0.3719	0.047*	
C4	0.3841 (3)	0.4185 (3)	0.3082 (2)	0.0345 (5)	
C5	0.3692 (5)	0.3305 (3)	0.2238 (3)	0.0555 (8)	
H5A	0.2763	0.2381	0.2182	0.067*	
C6	0.4886 (4)	0.3746 (3)	0.1456 (2)	0.0592 (9)	
H6A	0.4754	0.3125	0.0886	0.071*	
C7	0.2519 (4)	0.3655 (3)	0.3915 (2)	0.0427 (6)	
H7A	0.2849	0.4411	0.4457	0.051*	
H7B	0.1347	0.3536	0.3652	0.051*	
C8	0.2399 (3)	0.2229 (2)	0.43422 (17)	0.0251 (4)	
C9	0.1213 (3)	−0.1356 (3)	1.02159 (19)	0.0388 (6)	
C10	0.2361 (4)	0.0088 (3)	1.0244 (2)	0.0555 (8)	
H10A	0.2437	0.0702	1.0810	0.067*	
C11	0.3413 (4)	0.0630 (3)	0.9425 (2)	0.0553 (8)	
H11A	0.4198	0.1616	0.9448	0.066*	
C12	0.3332 (3)	−0.0242 (3)	0.85785 (18)	0.0353 (5)	
C13	0.2135 (4)	−0.1682 (3)	0.8556 (2)	0.0459 (7)	
H13A	0.2035	−0.2289	0.7983	0.055*	
C14	0.1074 (4)	−0.2248 (3)	0.9369 (2)	0.0492 (7)	
H14A	0.0271	−0.3227	0.9342	0.059*	
C15	0.4615 (3)	0.0341 (4)	0.77382 (19)	0.0479 (7)	
H15A	0.5323	0.1378	0.7900	0.058*	
H15B	0.5426	−0.0173	0.7732	0.058*	
C16	0.3832 (3)	0.0212 (2)	0.66965 (16)	0.0242 (4)	

### Atomic displacement parameters (Å^2^)

**Table:**

	*U*^11^	*U*^22^	*U*^33^	*U*^12^	*U*^13^	*U*^23^
Ce1	0.01487 (7)	0.02446 (7)	0.01999 (7)	0.00608 (5)	0.00407 (4)	0.00347 (4)
N1	0.0315 (11)	0.0260 (9)	0.0429 (13)	0.0112 (8)	−0.0058 (9)	−0.0002 (8)
O1W	0.0236 (8)	0.0339 (9)	0.0607 (13)	0.0071 (7)	−0.0001 (8)	0.0178 (8)
O1	0.0624 (14)	0.0546 (12)	0.0442 (13)	−0.0094 (10)	0.0298 (11)	−0.0038 (9)
O2	0.0210 (8)	0.0335 (9)	0.0589 (12)	0.0109 (7)	0.0102 (8)	0.0174 (8)
O2W	0.0340 (9)	0.0529 (11)	0.0230 (9)	0.0243 (8)	−0.0033 (7)	−0.0057 (7)
O3	0.0194 (7)	0.0307 (8)	0.0331 (9)	0.0048 (6)	0.0083 (7)	0.0039 (6)
O3W	0.0785 (17)	0.0687 (16)	0.0513 (15)	0.0324 (13)	0.0073 (13)	−0.0031 (11)
O4	0.0586 (14)	0.0721 (14)	0.0248 (11)	−0.0171 (11)	0.0180 (10)	−0.0021 (9)
O5	0.0219 (7)	0.0368 (8)	0.0207 (8)	0.0087 (6)	0.0063 (6)	0.0011 (6)
O6	0.0219 (8)	0.0644 (11)	0.0236 (9)	0.0192 (8)	0.0032 (7)	0.0003 (8)
O7	0.0589 (13)	0.0358 (10)	0.0941 (18)	0.0202 (10)	−0.0261 (13)	−0.0266 (11)
O8	0.0283 (9)	0.0636 (12)	0.0410 (11)	0.0064 (9)	0.0079 (8)	0.0069 (9)
O9	0.0352 (10)	0.0339 (9)	0.0484 (11)	0.0092 (8)	0.0131 (8)	−0.0003 (8)
C1	0.0383 (14)	0.0394 (13)	0.0352 (15)	0.0037 (11)	0.0138 (11)	0.0057 (11)
C2	0.0344 (14)	0.0401 (14)	0.0386 (16)	−0.0028 (11)	0.0087 (12)	0.0015 (11)
C3	0.0422 (14)	0.0419 (14)	0.0296 (14)	0.0124 (11)	0.0068 (11)	0.0025 (10)
C4	0.0386 (13)	0.0295 (11)	0.0391 (14)	0.0157 (10)	0.0147 (11)	0.0126 (10)
C5	0.0596 (19)	0.0303 (13)	0.058 (2)	−0.0042 (13)	0.0274 (16)	0.0008 (12)
C6	0.067 (2)	0.0388 (15)	0.0484 (19)	−0.0057 (14)	0.0284 (16)	−0.0081 (13)
C7	0.0450 (15)	0.0391 (13)	0.0534 (17)	0.0241 (12)	0.0268 (13)	0.0209 (12)
C8	0.0193 (10)	0.0288 (10)	0.0257 (12)	0.0073 (8)	0.0025 (8)	0.0040 (8)
C9	0.0373 (14)	0.0454 (14)	0.0220 (13)	0.0026 (11)	0.0068 (10)	0.0028 (10)
C10	0.062 (2)	0.0504 (17)	0.0303 (16)	−0.0042 (14)	0.0157 (14)	−0.0092 (12)
C11	0.0573 (18)	0.0456 (16)	0.0322 (16)	−0.0143 (13)	0.0101 (13)	−0.0032 (12)
C12	0.0259 (12)	0.0538 (15)	0.0174 (12)	0.0054 (11)	0.0007 (9)	0.0040 (10)
C13	0.0522 (17)	0.0505 (16)	0.0240 (14)	0.0080 (13)	0.0093 (12)	−0.0070 (11)
C14	0.0559 (18)	0.0397 (14)	0.0325 (16)	−0.0034 (13)	0.0117 (13)	−0.0011 (11)
C15	0.0229 (12)	0.083 (2)	0.0223 (13)	0.0018 (12)	0.0036 (10)	0.0068 (13)
C16	0.0204 (10)	0.0303 (10)	0.0208 (11)	0.0085 (8)	0.0036 (8)	0.0009 (8)

### Geometric parameters (Å, °)

**Table:**

Ce1—O3^i^	2.4914 (14)	O6—C16	1.256 (2)
Ce1—O5^ii^	2.4955 (14)	C1—C6	1.370 (4)
Ce1—O2W	2.5444 (16)	C1—C2	1.371 (4)
Ce1—O2	2.5451 (16)	C2—C3	1.392 (4)
Ce1—O1W	2.5463 (16)	C2—H2A	0.9300
Ce1—O6	2.5546 (17)	C3—C4	1.381 (4)
Ce1—O9	2.6411 (18)	C3—H3A	0.9300
Ce1—O5	2.6717 (15)	C4—C5	1.360 (4)
Ce1—O8	2.692 (2)	C4—C7	1.512 (3)
Ce1—O3	2.7245 (15)	C5—C6	1.393 (4)
Ce1—C16	2.990 (2)	C5—H5A	0.9300
N1—O7	1.211 (3)	C6—H6A	0.9300
N1—O9	1.258 (3)	C7—C8	1.497 (3)
N1—O8	1.261 (3)	C7—H7A	0.9700
O1W—H1WA	0.847 (16)	C7—H7B	0.9700
O1W—H1WB	0.819 (17)	C9—C10	1.362 (4)
O1—C1	1.377 (3)	C9—C14	1.372 (4)
O1—H1A	0.8200	C10—C11	1.382 (4)
O2—C8	1.253 (2)	C10—H10A	0.9300
O2W—H2WA	0.831 (16)	C11—C12	1.369 (4)
O2W—H2WB	0.835 (17)	C11—H11A	0.9300
O3—C8	1.262 (3)	C12—C13	1.371 (4)
O3—Ce1^i^	2.4914 (14)	C12—C15	1.508 (3)
O3W—H3WA	0.846 (18)	C13—C14	1.383 (4)
O3W—H3WB	0.818 (18)	C13—H13A	0.9300
O4—C9	1.378 (3)	C14—H14A	0.9300
O4—H4A	0.8200	C15—C16	1.491 (3)
O5—C16	1.263 (2)	C15—H15A	0.9700
O5—Ce1^ii^	2.4955 (14)	C15—H15B	0.9700
			
O3^i^—Ce1—O5^ii^	179.31 (5)	C8—O3—Ce1	90.79 (12)
O3^i^—Ce1—O2W	81.58 (5)	Ce1^i^—O3—Ce1	117.31 (6)
O5^ii^—Ce1—O2W	97.77 (5)	H3WA—O3W—H3WB	101 (3)
O3^i^—Ce1—O2	111.16 (5)	C9—O4—H4A	109.5
O5^ii^—Ce1—O2	68.82 (5)	C16—O5—Ce1^ii^	148.67 (14)
O2W—Ce1—O2	74.31 (6)	C16—O5—Ce1	91.74 (12)
O3^i^—Ce1—O1W	98.50 (5)	Ce1^ii^—O5—Ce1	118.06 (6)
O5^ii^—Ce1—O1W	81.87 (5)	C16—O6—Ce1	97.49 (13)
O2W—Ce1—O1W	141.07 (6)	N1—O8—Ce1	96.80 (13)
O2—Ce1—O1W	138.12 (5)	N1—O9—Ce1	99.36 (13)
O3^i^—Ce1—O6	69.62 (5)	C6—C1—C2	119.9 (2)
O5^ii^—Ce1—O6	111.05 (5)	C6—C1—O1	121.8 (2)
O2W—Ce1—O6	138.36 (5)	C2—C1—O1	118.2 (2)
O2—Ce1—O6	88.46 (6)	C1—C2—C3	120.0 (2)
O1W—Ce1—O6	74.55 (6)	C1—C2—H2A	120.0
O3^i^—Ce1—O9	110.48 (5)	C3—C2—H2A	120.0
O5^ii^—Ce1—O9	69.02 (5)	C4—C3—C2	120.7 (2)
O2W—Ce1—O9	67.10 (6)	C4—C3—H3A	119.6
O2—Ce1—O9	116.63 (6)	C2—C3—H3A	119.6
O1W—Ce1—O9	76.79 (6)	C5—C4—C3	118.1 (2)
O6—Ce1—O9	150.94 (6)	C5—C4—C7	120.2 (2)
O3^i^—Ce1—O5	118.73 (5)	C3—C4—C7	121.7 (2)
O5^ii^—Ce1—O5	61.94 (6)	C4—C5—C6	122.1 (3)
O2W—Ce1—O5	143.60 (5)	C4—C5—H5A	119.0
O2—Ce1—O5	70.22 (5)	C6—C5—H5A	119.0
O1W—Ce1—O5	69.76 (5)	C1—C6—C5	119.2 (3)
O6—Ce1—O5	49.13 (5)	C1—C6—H6A	120.4
O9—Ce1—O5	123.16 (5)	C5—C6—H6A	120.4
O3^i^—Ce1—O8	66.61 (5)	C8—C7—C4	114.46 (18)
O5^ii^—Ce1—O8	113.06 (5)	C8—C7—H7A	108.6
O2W—Ce1—O8	77.36 (6)	C4—C7—H7A	108.6
O2—Ce1—O8	151.55 (6)	C8—C7—H7B	108.6
O1W—Ce1—O8	67.42 (6)	C4—C7—H7B	108.6
O6—Ce1—O8	115.06 (6)	H7A—C7—H7B	107.6
O9—Ce1—O8	47.31 (5)	O2—C8—O3	119.32 (19)
O5—Ce1—O8	137.13 (6)	O2—C8—C7	120.53 (19)
O3^i^—Ce1—O3	62.69 (6)	O3—C8—C7	120.11 (18)
O5^ii^—Ce1—O3	117.26 (5)	C10—C9—C14	120.0 (2)
O2W—Ce1—O3	66.25 (5)	C10—C9—O4	117.6 (2)
O2—Ce1—O3	48.50 (5)	C14—C9—O4	122.3 (2)
O1W—Ce1—O3	147.40 (6)	C9—C10—C11	119.4 (3)
O6—Ce1—O3	73.89 (5)	C9—C10—H10A	120.3
O9—Ce1—O3	133.34 (5)	C11—C10—H10A	120.3
O5—Ce1—O3	95.12 (5)	C12—C11—C10	121.8 (3)
O8—Ce1—O3	120.31 (5)	C12—C11—H11A	119.1
O3^i^—Ce1—C16	94.08 (5)	C10—C11—H11A	119.1
O5^ii^—Ce1—C16	86.60 (5)	C11—C12—C13	117.8 (2)
O2W—Ce1—C16	152.02 (6)	C11—C12—C15	120.5 (2)
O2—Ce1—C16	81.73 (6)	C13—C12—C15	121.6 (2)
O1W—Ce1—C16	66.86 (6)	C12—C13—C14	121.3 (2)
O6—Ce1—C16	24.61 (5)	C12—C13—H13A	119.3
O9—Ce1—C16	138.72 (6)	C14—C13—H13A	119.3
O5—Ce1—C16	24.99 (5)	C9—C14—C13	119.6 (3)
O8—Ce1—C16	126.30 (6)	C9—C14—H14A	120.2
O3—Ce1—C16	87.12 (5)	C13—C14—H14A	120.2
O7—N1—O9	121.7 (2)	C16—C15—C12	117.1 (2)
O7—N1—O8	122.0 (2)	C16—C15—H15A	108.0
O9—N1—O8	116.4 (2)	C12—C15—H15A	108.0
Ce1—O1W—H1WA	120 (2)	C16—C15—H15B	108.0
Ce1—O1W—H1WB	137 (2)	C12—C15—H15B	108.0
H1WA—O1W—H1WB	103 (2)	H15A—C15—H15B	107.3
C1—O1—H1A	109.5	O6—C16—O5	119.4 (2)
C8—O2—Ce1	99.57 (13)	O6—C16—C15	122.36 (19)
Ce1—O2W—H2WA	118.0 (19)	O5—C16—C15	118.19 (19)
Ce1—O2W—H2WB	130 (2)	O6—C16—Ce1	57.90 (11)
H2WA—O2W—H2WB	104 (2)	O5—C16—Ce1	63.27 (11)
C8—O3—Ce1^i^	151.47 (14)	C15—C16—Ce1	164.79 (18)
			
O3^i^—Ce1—O2—C8	−9.94 (16)	O1W—Ce1—O9—N1	73.79 (13)
O5^ii^—Ce1—O2—C8	169.33 (16)	O6—Ce1—O9—N1	64.17 (18)
O2W—Ce1—O2—C8	64.29 (14)	O5—Ce1—O9—N1	128.49 (13)
O1W—Ce1—O2—C8	−141.93 (14)	O8—Ce1—O9—N1	2.46 (12)
O6—Ce1—O2—C8	−77.35 (15)	O3—Ce1—O9—N1	−91.75 (14)
O9—Ce1—O2—C8	117.92 (14)	C16—Ce1—O9—N1	102.24 (14)
O5—Ce1—O2—C8	−124.05 (15)	C6—C1—C2—C3	−0.1 (5)
O8—Ce1—O2—C8	69.7 (2)	O1—C1—C2—C3	179.4 (3)
O3—Ce1—O2—C8	−7.65 (13)	C1—C2—C3—C4	0.4 (4)
C16—Ce1—O2—C8	−101.13 (15)	C2—C3—C4—C5	−0.4 (4)
O3^i^—Ce1—O3—C8	−174.91 (17)	C2—C3—C4—C7	179.4 (2)
O5^ii^—Ce1—O3—C8	4.31 (15)	C3—C4—C5—C6	0.2 (5)
O2W—Ce1—O3—C8	−82.21 (13)	C7—C4—C5—C6	−179.7 (3)
O2—Ce1—O3—C8	7.49 (13)	C2—C1—C6—C5	−0.1 (5)
O1W—Ce1—O3—C8	124.99 (14)	O1—C1—C6—C5	−179.6 (3)
O6—Ce1—O3—C8	110.09 (14)	C4—C5—C6—C1	0.1 (6)
O9—Ce1—O3—C8	−81.81 (14)	C5—C4—C7—C8	57.1 (4)
O5—Ce1—O3—C8	65.30 (13)	C3—C4—C7—C8	−122.7 (3)
O8—Ce1—O3—C8	−139.93 (13)	Ce1—O2—C8—O3	14.3 (2)
C16—Ce1—O3—C8	88.99 (13)	Ce1—O2—C8—C7	−163.7 (2)
O3^i^—Ce1—O3—Ce1^i^	0.0	Ce1^i^—O3—C8—O2	176.4 (2)
O5^ii^—Ce1—O3—Ce1^i^	179.23 (5)	Ce1—O3—C8—O2	−13.1 (2)
O2W—Ce1—O3—Ce1^i^	92.70 (8)	Ce1^i^—O3—C8—C7	−5.7 (5)
O2—Ce1—O3—Ce1^i^	−177.60 (10)	Ce1—O3—C8—C7	164.8 (2)
O1W—Ce1—O3—Ce1^i^	−60.09 (12)	C4—C7—C8—O2	24.1 (4)
O6—Ce1—O3—Ce1^i^	−75.00 (7)	C4—C7—C8—O3	−153.9 (2)
O9—Ce1—O3—Ce1^i^	93.10 (8)	C14—C9—C10—C11	−1.7 (5)
O5—Ce1—O3—Ce1^i^	−119.79 (7)	O4—C9—C10—C11	175.2 (3)
O8—Ce1—O3—Ce1^i^	34.98 (9)	C9—C10—C11—C12	0.2 (6)
C16—Ce1—O3—Ce1^i^	−96.10 (7)	C10—C11—C12—C13	1.4 (5)
O3^i^—Ce1—O5—C16	10.28 (14)	C10—C11—C12—C15	−174.3 (3)
O5^ii^—Ce1—O5—C16	−169.91 (16)	C11—C12—C13—C14	−1.5 (5)
O2W—Ce1—O5—C16	127.78 (12)	C15—C12—C13—C14	174.0 (3)
O2—Ce1—O5—C16	114.18 (13)	C10—C9—C14—C13	1.5 (5)
O1W—Ce1—O5—C16	−78.44 (13)	O4—C9—C14—C13	−175.2 (3)
O6—Ce1—O5—C16	8.32 (12)	C12—C13—C14—C9	0.1 (5)
O9—Ce1—O5—C16	−136.31 (12)	C11—C12—C15—C16	−127.4 (3)
O8—Ce1—O5—C16	−75.42 (14)	C13—C12—C15—C16	57.1 (4)
O3—Ce1—O5—C16	71.84 (12)	Ce1—O6—C16—O5	15.6 (2)
O3^i^—Ce1—O5—Ce1^ii^	−179.81 (5)	Ce1—O6—C16—C15	−162.0 (2)
O5^ii^—Ce1—O5—Ce1^ii^	0.0	Ce1^ii^—O5—C16—O6	−177.51 (17)
O2W—Ce1—O5—Ce1^ii^	−62.31 (11)	Ce1—O5—C16—O6	−14.8 (2)
O2—Ce1—O5—Ce1^ii^	−75.91 (7)	Ce1^ii^—O5—C16—C15	0.2 (4)
O1W—Ce1—O5—Ce1^ii^	91.47 (8)	Ce1—O5—C16—C15	162.9 (2)
O6—Ce1—O5—Ce1^ii^	178.23 (10)	Ce1^ii^—O5—C16—Ce1	−162.7 (3)
O9—Ce1—O5—Ce1^ii^	33.60 (9)	C12—C15—C16—O6	12.9 (4)
O8—Ce1—O5—Ce1^ii^	94.49 (9)	C12—C15—C16—O5	−164.8 (2)
O3—Ce1—O5—Ce1^ii^	−118.25 (7)	C12—C15—C16—Ce1	−73.2 (6)
C16—Ce1—O5—Ce1^ii^	169.91 (16)	O3^i^—Ce1—C16—O6	−6.21 (14)
O3^i^—Ce1—O6—C16	173.39 (15)	O5^ii^—Ce1—C16—O6	173.67 (14)
O5^ii^—Ce1—O6—C16	−6.77 (15)	O2W—Ce1—C16—O6	73.49 (19)
O2W—Ce1—O6—C16	−137.39 (13)	O2—Ce1—C16—O6	104.60 (14)
O2—Ce1—O6—C16	−73.33 (14)	O1W—Ce1—C16—O6	−103.72 (15)
O1W—Ce1—O6—C16	67.94 (14)	O9—Ce1—C16—O6	−134.01 (14)
O9—Ce1—O6—C16	77.67 (18)	O5—Ce1—C16—O6	164.8 (2)
O5—Ce1—O6—C16	−8.44 (12)	O8—Ce1—C16—O6	−70.02 (15)
O8—Ce1—O6—C16	123.27 (14)	O3—Ce1—C16—O6	56.13 (14)
O3—Ce1—O6—C16	−120.32 (14)	O3^i^—Ce1—C16—O5	−170.98 (12)
O7—N1—O8—Ce1	−176.2 (2)	O5^ii^—Ce1—C16—O5	8.91 (14)
O9—N1—O8—Ce1	4.2 (2)	O2W—Ce1—C16—O5	−91.28 (16)
O3^i^—Ce1—O8—N1	154.13 (15)	O2—Ce1—C16—O5	−60.17 (12)
O5^ii^—Ce1—O8—N1	−25.22 (15)	O1W—Ce1—C16—O5	91.51 (12)
O2W—Ce1—O8—N1	67.94 (13)	O6—Ce1—C16—O5	−164.8 (2)
O2—Ce1—O8—N1	62.56 (19)	O9—Ce1—C16—O5	61.22 (15)
O1W—Ce1—O8—N1	−95.12 (14)	O8—Ce1—C16—O5	125.22 (12)
O6—Ce1—O8—N1	−154.27 (12)	O3—Ce1—C16—O5	−108.63 (12)
O9—Ce1—O8—N1	−2.44 (12)	O3^i^—Ce1—C16—C15	89.6 (6)
O5—Ce1—O8—N1	−98.19 (14)	O5^ii^—Ce1—C16—C15	−90.5 (6)
O3—Ce1—O8—N1	120.41 (13)	O2W—Ce1—C16—C15	169.3 (5)
C16—Ce1—O8—N1	−128.67 (13)	O2—Ce1—C16—C15	−159.6 (6)
O7—N1—O9—Ce1	176.07 (19)	O1W—Ce1—C16—C15	−7.9 (5)
O8—N1—O9—Ce1	−4.3 (2)	O6—Ce1—C16—C15	95.8 (6)
O3^i^—Ce1—O9—N1	−20.48 (15)	O9—Ce1—C16—C15	−38.2 (6)
O5^ii^—Ce1—O9—N1	160.03 (15)	O5—Ce1—C16—C15	−99.5 (6)
O2W—Ce1—O9—N1	−91.36 (14)	O8—Ce1—C16—C15	25.8 (6)
O2—Ce1—O9—N1	−148.66 (12)	O3—Ce1—C16—C15	151.9 (6)

Symmetry codes: (i) −*x*, −*y*, −*z*+1; (ii) −*x*+1, −*y*, −*z*+1.

### Hydrogen-bond geometry (Å, °)

**Table:**

*D*—H···*A*	*D*—H	H···*A*	*D*···*A*	*D*—H···*A*
O1W—H1WA···O2^ii^	0.85 (2)	1.90 (2)	2.693 (2)	156 (3)
O1W—H1WB···O7^iii^	0.82 (2)	2.46 (2)	3.197 (3)	150 (3)
O1W—H1WB···O8^iii^	0.82 (2)	2.51 (2)	3.272 (3)	156 (3)
O2W—H2WA···O6^i^	0.83 (2)	1.91 (2)	2.721 (2)	165 (3)
O2W—H2WB···O4^iv^	0.84 (2)	1.95 (2)	2.783 (3)	175 (3)
O3W—H3WA···O4^ii^	0.85 (2)	2.58 (2)	3.352 (4)	152 (3)
O4—H4A···O1^v^	0.82	1.83	2.649 (3)	173

Symmetry codes: (ii) −*x*+1, −*y*, −*z*+1; (iii) −*x*, −*y*−1, −*z*+1; (i) −*x*, −*y*, −*z*+1; (iv) *x*, *y*, *z*−1; (v) *x*−1, *y*−1, *z*+1.
